# Prognostic significance of glucose‐lipid metabolic index in pancreatic cancer patients with diabetes mellitus

**DOI:** 10.1002/cam4.7108

**Published:** 2024-03-25

**Authors:** Hailiang Wang, Shiye Ruan, Zelong Wu, Qian Yan, Yubin Chen, Jinwei Cui, Zhongyan Zhang, Shanzhou Huang, Baohua Hou, Chuanzhao Zhang

**Affiliations:** ^1^ Department of General Surgery, Guangdong Provincial People's Hospital (Guangdong Academy of Medical Sciences) Southern Medical University Guangzhou China; ^2^ Department of Hepatobiliary Surgery Weihai Central Hospital, Qingdao University Weihai China; ^3^ The Second School of Clinical Medicine Southern Medical University Guangzhou China; ^4^ School of Medicine South China University of Technology Guangzhou China; ^5^ Department of General Surgery Heyuan People's Hospital Heyuan China

**Keywords:** diabetes mellitus, glucose‐lipid metabolism index, insulin resistance, pancreatic cancer, prognosis

## Abstract

**Background:**

The incidence of pancreatic cancer (PC) is higher in diabetic patients due to disturbances in glucose and lipid metabolism caused by insulin resistance (IR). However, the effect of diabetes as well as IR on the prognosis of PC patients remains inconclusive. Our study aims to assess the impact of IR on the prognosis of PC patients with diabetes.

**Methods:**

We conducted a retrospective analysis of 172 PC patients with diabetes in our institute from 2015 to 2021. Prognostic assessment was performed using univariate/multifactorial analysis and survival analysis. The predictive efficacy of metabolic indices was compared using receiver operator characteristic (ROC) curve analysis.

**Results:**

One hundred twenty‐one of 172 patients died during follow‐up, with a median follow‐up of 477 days and a median overall survival (OS) of 270 days. Survival analysis showed a significant difference in OS by IR related parameters, which were triglyceride‐glucose index (TyG), triglyceride‐glucose index‐body mass index (TyG‐BMI), and triglyceride/high‐density lipoprotein cholesterol ratio (TG/HDL‐c). The ROC curve indicated that TyG, TyG‐BMI, and TG/HDL‐c had prognostic efficacy for PC with diabetes. We next optimized TyG‐BMI and obtained a new parameter, namely glucose‐lipid metabolism index (GLMI), and the patients were classified into GLMI low group and high group based on the calculated cutoff value. The GLMI high group had higher TyG, TyG‐BMI, TyG/HDL‐c, BMI, TG, total cholesterol (TC), TC/HDL‐c, fasting plasma glucose, CA199, and more advanced tumor stage compared to low group. Univariate and multivariate analyses showed that GLMI was an independent prognostic factor. Furthermore, the patients of GLMI high group had worse OS compared to low group and the ROC curves showed GLMI had better predictive ability than TyG and TyG‐BMI.

**Conclusions:**

IR is associated with the outcome of PC patients with diabetes and higher level of IR indicates worse prognosis. GLMI has a good predictive value for PC with diabetes.

## Graphical abstract

The incidence of pancreatic cancer (PC) is higher in people with diabetes mellitus due to disturbances in glucose and lipid metabolism caused by insulin resistance (IR). However, the effect of diabetes mellitus as well as IR on the prognosis of PC patients remains inconclusive. Our study aims to assess the impact of IR on the prognosis of PC patients with diabetes mellitus.

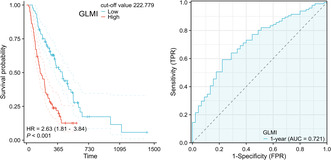

## 1 INTRODUCTION

Pancreatic cancer (PC) is one of the deadliest cancers because of the difficulties in early detection and treatment. PC's prognosis remains discouraging with a 5‐year relative survival rate of only 5%; and, it is predicted to become the second leading cause of cancer‐related fatalities.^1^, ^2^ Although efforts were made by clinicians and researchers, there has been little significant improvement in early diagnosis and survival of PC in recent decades.^3^ Therefore, investigation of biological and clinical features, as well as biomarkers for PC are very important for discovery of better treating strategy.

There is growing evidence that diabetes is a high risk factor for PC incidence,^3^, ^4^, ^5^, ^6^ and patients with diabetes have a 1.5‐ to 2‐fold increased risk of developing PC.^7^ Nevertheless, the impact of diabetes on the prognosis of PC remains uncertain. Several studies have reported on a worse prognosis for PC patients with diabetes, compared to non‐diabetic PC patients.^8^, ^9^, ^10^, ^11^, ^12^, ^13^ These results emphasize PC patients with diabetes is a special population with high risk. We previously found fasting blood glucose and inflammation factors were risk factor for PC patients with diabetes (in publication process). Diabetes mellitus is a metabolic disorder and has the characteristics of decreased insulin secretion and/or insulin resistance (IR).^1^, ^14^ According to existed researches, IR increases the risk of various types of cancer, such as prostate cancer,^15^ non‐small cell lung cancer,^16^ gastric cancer,^17^ and obesity‐related cancers.^18^ And it is also associated with a higher mortality rate.^19^ However, it remains unclear whether IR is correlated with patients' prognosis for PC patients with diabetes.

In this study, we aim at evaluating the predictive value of IR in the prognosis of PC patients with diabetes mellitus by analyzing triglyceride‐glucose index (TyG), triglyceride/high‐density lipoprotein cholesterol ratio (TG/HDL‐c), and triglyceride‐glucose index‐body mass index (TyG‐BMI), which were simple measures of IR.^20^, ^21^, ^22^, ^23^, ^24^, ^25^ We also develop a modified index, which is glucose‐lipid metabolism index (GLMI) and test its predictive ability in PC patients with diabetes mellitus.

## 2 MATERIALS AND METHODS

### 2.1 Clinical information

**FIGURE 1:**
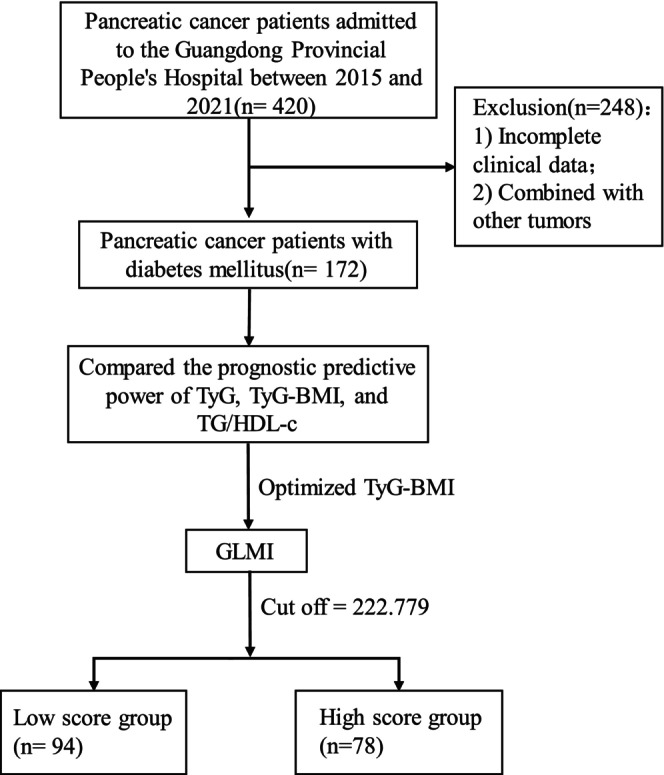
Patient flow diagram.

Figure 1 depicts a flow diagram of the patient cohort. The clinical data of 420 PC patients admitted to the Pancreatic Center of Guangdong Provincial People's Hospital between 2015 and 2021 was retrospectively analyzed in this study. The inclusion criteria for this study were: (1) Preliminary imaging diagnosis and pathological examination of PC, and (2) presence of diabetes mellitus or impaired glucose tolerance (IGT). The diagnostic criteria for diabetes mellitus in this study were: fasting plasma glucose (FPG) level of ≥7.0 mmol/L. Additionally, diagnostic criteria for diabetes mellitus included a 75 g oral glucose tolerance test (OGTT) with 2 h FPG level of ≥11.1 mmol/L, or glycated hemoglobin >6.5%, or previous diagnosis of diabetes or use of hypoglycemic agents. Criteria for IGT were a 75 g OGTT with 2‐h FPG level ≥7.8 mmol/L.^26^ The exclusion criteria were: These included incomplete clinical and pathological data and the presence of combined other tumors. A total of 172 PC patients with diabetes mellitus were included in the follow‐up analysis. The Ethics Committee of the Guangdong Academy of Medical Sciences approved this study.

Demographic, serologic, radiologic, and pathologic information was collected from eligible patients. Such as age, sex, body mass index (BMI), triglycerides (TG), total cholesterol (TC), high‐density lipoprotein cholesterol (HDL‐c), TC/HDL‐c, FPG, hemoglobin (HB), total bilirubin (TBIL), alanine aminotransferase (ALT), γ‐glutamyl transpeptidase (γ‐GGT), albumin (ALB), activated partial thromboplastin time (APTT), CA125, CEA, CA19‐9, treatment and TNM staging. TNM staging was performed according to the 8th edition of the AJCC PC staging system. Based on the above indicators, TyG, TyG‐BMI, TG/HDL‐c and GLMI were calculated, respectively, and the calculation methods were as follows^27^, ^28^:

$$\mathrm{TyG}=\ln\;\left[\mathrm{TG}\;\left(\mathrm{mg}/\mathrm{dL}\right)\times \mathrm{FPG}\;\left(\mathrm{mg}/\mathrm{dL}\right)/2\right];$$

$$\mathrm{TG}/\mathrm{HDL}‐c=\mathrm{TG}\;\left(\mathrm{mg}/\mathrm{dL}\right)\times \mathrm{HDL}\;\left(\mathrm{mg}/\mathrm{dL}\right);$$

$$\mathrm{BMI}=\mathrm{Weight}\;\left(\mathrm{kg}\right)/{\text{Height}}^{2}\;\left(m^{2}\right)\;\left(\mathrm{kg}/m^{2}\right);$$

TyG‐BMI=TyG×BMI;

GLMI=TyG‐BMI×TC/HDL‐c.

All patients who were enrolled underwent an outpatient and telephone follow‐up along with a review of their medical history. The primary endpoint was the OS, which was defined as the duration between diagnosis and either death or the last follow‐up. The last follow‐up was accomplished on July 31, 2022.

### 2.2 Statistical methods

The statistical analysis was performed using SPSS 27.0 software. The clinicopathological data of the patients were presented as mean ± standard deviation for continuous variables and frequency (percentage) for categorical variables. For continuous variables, independent samples *t*‐test was carried out whereas for categorical variables, chi‐squared test/Fisher exact test was performed. COX proportional hazards regression analysis was conducted to evaluate the univariate and multivariate survival risk. Kaplan–Meier analysis (log‐rank test) was utilized to plot the survival curves, and to determine the optimal survival‐related cutoff value for GLMI. Furthermore, the predictive efficacy of each metabolic index was compared using time‐dependent receiver operator characteristic (ROC) curve analysis to calculate the area under the curve (AUC). *p* < 0.05 (two‐tailed) differences were considered statistically significant.

## 3 RESULTS

The study included 172 PC patients with diabetes mellitus, who were ultimately followed for a median of 477 days. Out of these, 121 patients died within the follow‐up period with a median survival of 270 days.

### 3.1 Evaluation of the prognostic value of TyG, TyG‐BMI, TG/HDL‐c in PC patients with diabetes

**FIGURE 2:**
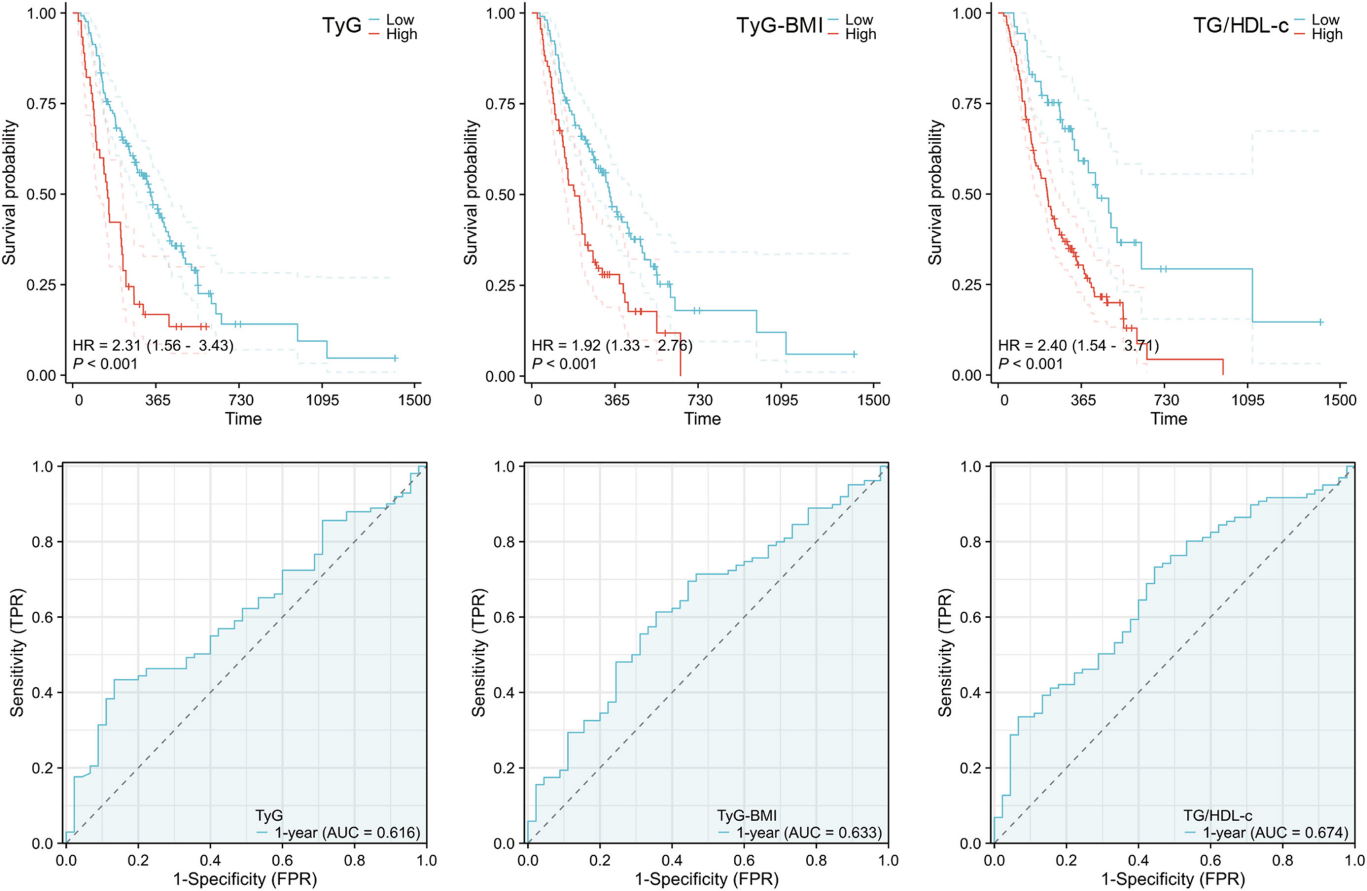
K–M curves for overall survival by different IR indexes and receiver operator characteristic curves for different IR indexes. IR, insulin resistance.

To find out whether IR is associated with patients' prognosis, we first investigate the correlation between IR related parameters and patients' survival, which were TyG, TyG‐BMI and TG/HDL‐c. We set up the cutoff points for these three parameters and performed Kaplan–Meier survival analysis. The results showed a significant difference in OS by TyG (HR = 2.231; 95% CI: 1.56–3.43; *p* < 0.001), TyG‐BMI (HR = 1.92; 95% CI: 1.33–2.76; *p* < 0.001), TG/HDL‐c (HR = 2.40; 95% CI: 1.54–3.71; *p* < 0.001) (Figure 2). Time‐dependent ROC curve showed that TyG (AUC = 0.616, 95% CI: 0.5218–0.7099), TyG‐BMI (AUC = 0.633, 95% CI: 0.537–0.7284), TG/HDL‐c (AUC = 0.674, 95% CI: 0.5818–0.7663) had prognostic efficacy, although not a very high level, in PC patients with diabetes (Figure 2).

### 3.2 GLMI, a new parameter, is associated with IR and tumor stage in PC patients with diabetes mellitus

In order to achieve a superior predictive value, we optimized TyG‐BMI and obtained GLMI (GLMI = TyG × BMI × TC/HDL‐c). Using Kaplan–Meier survival curves, the optimal cutoff value for the GLMI was calculated to be 222.779. Based on this cutoff value, patients were classified into two groups: a low IR score group (low group) and a high IR score group (high group). GLMI scores ≤222.779 were used as the low group, which had 94 cases (54.7%), and GLMI scores >222.779 were used as the high group, which had 78 cases (45.3%). The high group had significantly higher TyG, TyG‐BMI, TyG/HDL‐c, BMI, TG, TC, TC/HDL‐c, FPG, HDL‐c, CA199, HB, ALB, TBIL, and more advanced TNM stage compared to the low group. In stage I and II, the number of patients in the GLMI high group was significantly lower than that in the low group (33 vs. 56 patients); however, in stage III and IV, the number of patients in the GLMI high group was significantly higher than that in the low group (45 vs. 38 patients). The statistical difference was significant (*p* < 0.05). No statistically significant differences were observed in age, gender, treatment, location, CEA, CA125, γ‐GGT, ALT, and APTT between the groups (*p* > 0.05) (Table 1). These indicate higher GLMI is associated with IR and advanced tumor stage in PC patients with diabetes mellitus.

**TABLE 1:** Baseline data from 172 pancreatic cancer patients with diabetes mellitus with varying degrees of insulin resistance.

Variable	Total	Low	High	*p* Value
		*N* = 94 (54.7%)	*N* = 78 (45.3%)	
Age		60.35 ± 11.10	62.56 ± 11.27	0.198
Gender				0.514
Male	99 (57.6%)	52 (30.2%)	47 (27.3%)	
Female	73 (42.4%)	42 (24.4%)	31 (18%)	
Treatment				0.102
Operation	105 (61.1%)	63 (36.6%)	42 (24.4%)	
Chemotherapy	48 (27.9%)	20 (11.6%)	28 (16.3%)	
Others	19 (11.0%)	11 (6.4%)	8 (4.7%)	
TNM stage				0.024*
I and II	89 (51.7%)	56 (32.6%)	33 (19.2%)	
III and IV	83 (48.3%)	38 (22.1%)	45 (26.2%)	
Location				0.832
Head	111 (64.5%)	60 (34.9%)	51 (29.7%)	
Corpus and cauda	61 (35.5%)	34 (19.8%)	27 (15.7%)	
BMI		20.56 ± 2.61	22.36 ± 2.70	<0.001*
TG (mmol/L)		1.42 ± 0.64	2.82 ± 1.14	<0.001*
TC (mmol/L)		4.34 ± 1.08	5.42 ± 1.41	<0.001*
TC/HDL‐c		4.01 ± 1.09	6.84 ± 2.43	<0.001*
HDL‐c (mmol/L)		1.13 ± 0.30	0.86 ± 0.28	<0.001*
FPG (mmol/L)		8.87 ± 2.41	10.99 ± 3.87	<0.001*
CEA (ng/mL)		13.29 ± 43.62	27.15 ± 103.55	0.240
CA199 (U/mL)		376.03 ± 402.36	521.38 ± 445.28	0.026*
CA125 (U/mL)		107.68 ± 413.51	100.00 ± 172.88	0.878
HB (g/L)		124.16 ± 16.57	118.67 ± 16.85	0.033*
ALB (g/L)		37.93 ± 3.30	35.70 ± 4.42	0.013*
γ‐GGT (U/L)		298.86 ± 465.80	380.63 ± 446.55	0.245
ALT (U/L)		97.77 ± 134.26	92.87 ± 124.07	0.806
TBIL (μmol/L)		67.91 ± 97.10	117.42 ± 143.99	<0.011*
APTT (s)		37.08 ± 4.53	37.61 ± 5.26	0.48
TyG		1.72 ± 0.50	2.61 ± 0.47	<0.001*
TyG‐BMI		35.32 ± 11.43	58.31 ± 12.72	<0.001*
TG/HDL‐c		1.32 ± 0.67	3.58 ± 1.76	<0.001*

Abbreviations: ALB, albumin; ALT, alanine aminotransferase; APTT, activated partial thromboplastin time; BMI, body mass index; FPG, fasting plasma glucose; HB, hemoglobin; HDL‐c, high‐density lipoprotein cholesterol; TBIL, total bilirubin; TC, total cholesterol; TG, triglycerides; TyG, triglyceride‐glucose index; TyG‐BMI, triglyceride‐glucose index‐body mass index; γ‐GGT, γ‐glutamyl transpeptidase.

* *p* < 0.05.

### 3.3 GLMI is an independent risk factor for PC patients with diabetes mellitus

Univariate analysis showed that high GLMI (>222.779) (HR = 2.781; 95% CI: 1.908–4.053; *p* < 0.001), location (HR = 0.688; 95% CI: 0.477–0.992; *p* = 0.045), age (HR = 1.020; 95% CI: 1.001–1.038; *p* = 0.034), TNM stage (HR = 3.056; 95% CI: 2.101–4.445; *p* < 0.001), treatment (HR = 2.096; 95% CI: 1.645–2.670; *p* < 0.001), CA199 (HR = 1.001; 95% CI: 1.000–1.001; *p* = 0.008), CA125 (HR = 1.000; 95% CI: 1.000–1.001; *p* = 0.002), and CEA (HR = 1. 004; 95% CI: 1.002–1.006; *p* < 0.001) were significant prognostic factors in PC patients with diabetes. Furthermore, multivariate analysis was performed and the results suggested that high GLMI (HR = 2.696; 95% CI: 1.828–3.976; *p* < 0.001), TNM stage (HR = 1.719; 95% CI: 1.020–2.898; *p =* 0.042), CEA (HR = 1.002; 95% CI: 1.000–1.004; *p* = 0.016) and treatment (HR = 1.628; 95% CI: 1.121–2.366; *p* = 0.011) were independent prognostic factors in PC patients with diabetes mellitus (Table 2).

**TABLE 2:** Univariate and multivariate analysis of overall survival in 172 patients with pancreatic cancer and diabetes.

Variable	Total	Univariate analysis				Multivariate analysis			
		HR	95% CI		*p* Value	HR	95% CI		*p* Value
			Lower	Upper			Lower	Upper	
GLMI									
High >222.779	78	2.781	1.908	4.053	<0.001*	2.696	1.828	3.976	<0.001*
Low ≤222.779	94								
Gender									
Male	99	0.832	0.579	1.197	0.322				
Female	73								
Age		1.020	1.001	1.038	0.034*				
Location									
Head and neck	111	0.688	0.477	0.992	0.045*				
Body and tail	61								
TNM stage									
I and II	89	3.056	2.101	4.445	<0.001*	1.719	1.020	2.898	0.042*
III and IV	83								
Treatment									
Operation	105	2.096	1.645	2.670	<0.001*	1.628	1.121	2.366	0.011*
Chemotherapy	48								
Others	19								
CA199		1.001	1.000	1.001	0.008*				
CA125		1.001	1.000	1.001	0.002*				
CEA		1.004	1.002	1.006	<0.001*	1.002	1.000	1.004	0.016*
HB		0.998	0.987	1.008	0.678				
ALB		0.979	0.937	1.024	0.362				
ALT		1.000	0.998	1.001	0.693				
γ‐GGT		1.000	0.999	1.000	0.507				
TBIL		1.000	0.998	1.001	0.894				
APTT		0.988	0.950	1.027	0.535				

Abbreviations: ALB, albumin; ALT, alanine aminotransferase; APTT, activated partial thromboplastin time; GLMI, glucose‐lipid metabolism index; HB, hemoglobin; TBIL, total bilirubin; γ‐GGT, γ‐glutamyl transpeptidase.

* *p* < 0.05.

### 3.4 The predictive value of GLMI for patients' survival in PC with diabetes

**FIGURE 3:**
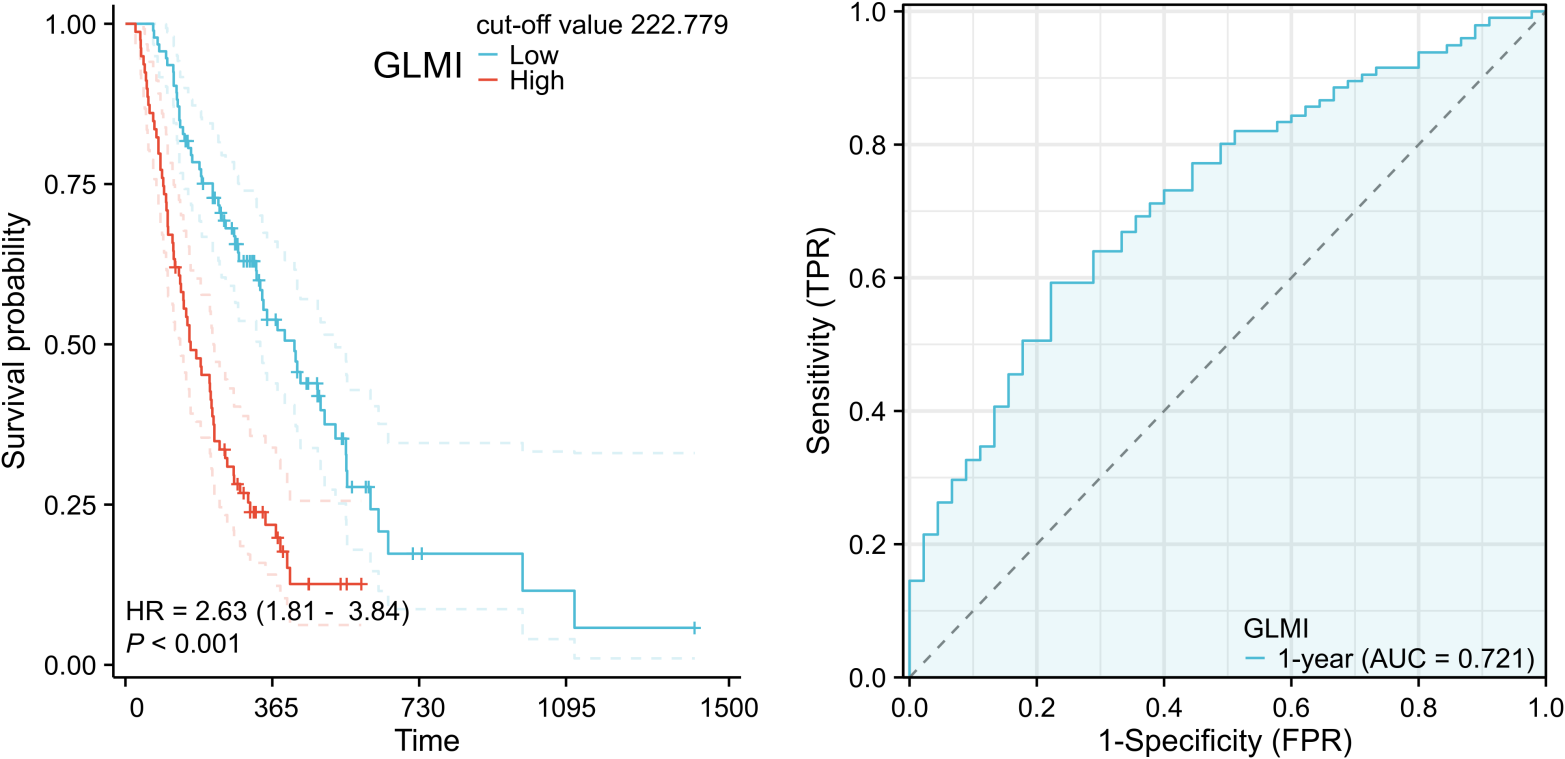
K–M curves for overall survival by different GLMI level and receiver operator characteristic curves for GLMI. GLMI, glucose‐lipid metabolism index.

Kaplan–Meier survival curves showed a significant difference in OS by GLMI (HR = 2.63; 95% CI: 1.81–3.84; *p* < 0.001) (Figure 3). Indeed, the median OS between GLMI high group (78 patients) and low group (94 patients) were 162 days (95% CI: 136–219 days) versus 420 days (95% CI: 335–522 days, *p* < 0.001). GLMI exhibited greater prognostic efficacy (AUC = 0.721, 95% CI: 0.6349–0.8085) (Figure 3) in comparison to TyG (*p* = 0.0035), TyG‐BMI (*p* = 0.0126), and exhibited no significant difference as compared to TG/HDL‐c (*p* = 0.1126).

## 4 DISCUSSION

Diabetes is associated with increased incidence of PC. As economic levels have risen and dietary structures have changed, the prevalence of diabetes mellitus has gradually increased, alongside increased incidence of PC.^29^, ^30^ In 50%–80% of PC patients, diabetes mellitus is comorbid,^31^, ^32^ and approximately 85% have IGT.^33^ However, several meta‐analyses on the effect of diabetes mellitus on the prognosis of PC have reached contradictory conclusions.^8^, ^9^, ^11^, ^12^, ^13^, ^34^, ^35^ Some studies found diabetes led to worse survival compared to non‐diabetes PC patients. Indeed, hyperglycemia may promote malignant phenotype of cancer cells. For example, hyperglycemia fosters tumor cell growth by creating a nutrient‐rich environment and by promoting the resistance of PC cells to gemcitabine through the regulation of the ROS/MMP‐3 signaling pathway.^36^ Moreover, hyperglycemia fuels the proliferation of PC cells through the induction of EGF expression and EGFR activation.^37^ In contrast, a few studies found comparable prognosis between PC patients with or without diabetes. The different results may be attributed to the different glucose controlling level or administration of anti‐hyperglycemic medications like metformin.^38^ Further studies are needed to investigate the influence of glucose controlling level and oral hypoglycemic drugs on cancer‐related outcome.

Insulin resistance plays a crucial role in the pathophysiology of diabetes mellitus and causes a variety of metabolic disorders such as hyperglycemia, hyperinsulinemia, dyslipidemia and visceral obesity.^16^, ^26^, ^28^, ^39^, ^40^ Currently, the homeostatic model assessment of IR and the hyperinsulinemic euglycemic clamp are accurate methods for assessing IR. However, due to their complexity, high cost of operation, and lack of a standardized method for insulin determination, these two methods are not favorable for clinical promotion.^27^, ^28^, ^41^ Three metabolic indices, namely TyG, TyG‐BMI, and TG/HDL‐c, have been identified as reliable indicators for assessing the degree of IR due to their simplicity and practicality.^26^, ^27^, ^28^, ^41^ The time‐dependent ROC curves demonstrated that TyG (AUC = 0.616), TG/HDL‐c (AUC = 0.674), and TyG‐BMI (AUC = 0.633) all had predictive value for the prognosis of PC patients with diabetes mellitus. Other studies also found IR occurred in 85%–95% of all diabetic patients^30^, ^42^ and was associated with the aggressiveness of pancreatic ductal adenocarcinoma.^43^ Research has shown that diabetic patients face a higher risk of cancer recurrence and cancer‐related death.^11^ Mechanistically, high insulin levels can promote the proliferation and differentiation of PC cells and angiogenesis through the activation of the insulin and insulin‐like growth factor axis^44^ and the PI3K/Akt and MAPK signaling pathways,^30^, ^42^ leading to PC progression. In addition, IR can induce hyperglycemia, hyperinsulinemia, and dyslipidemia, which may promote PC cells to remodel their metabolic phenotypes and increase their aggressiveness.^1^, ^45^ Combining these with our findings, we believe that IR is a critical parameter which affects outcome of PC patients with diabetes.

The predictive efficacy of TG/HDL‐c (AUC = 0.674) was found to be superior to that of TyG (AUC = 0.616) and TyG‐BMI (AUC = 0.633), as depicted in Figure 2. Contrary to our findings, in studies of benign diseases, TyG‐BMI and TyG showed superior efficacy in evaluating IR than TG/HDL‐c.^27^, ^28^ We speculated that cholesterol metabolism may affect the progression of PC, as IR results in elevated triglyceride levels and decreased HDL‐c levels.^46^ And cholesterol metabolism is connected to all stages of tumors, including tumorigenesis, tumor resistance, and immune escape. These effects are primarily exerted through regulating the structure and function of cell membranes, cellular homeostasis, and hormone synthesis.^47^, ^48^, ^49^ Therefore, with reference to the optimization of TyG‐neck circumference (TyG‐NC), TyG‐neck circumference to height ratio (TyG‐NHtR), TyG‐waist circumference (TyG‐WC), TyG‐waist to height ratio (TyG‐WHtR), TyG‐body mass index (TyG‐BMI),^22^ we optimized TyG‐BMI, and the product of TyG‐BMI and TC/HDL‐c was used as the GLMI. The level of IR in PC patients with diabetes was evaluated using GLMI. The Kaplan–Meier survival curves demonstrated that patients with a high GLMI score had a median OS of 162 days while the low score group had 420 days (*p* < 0.001). Our analysis revealed that GLMI was an independent prognostic factor for PC patients with diabetes mellitus, confirmed by both univariate and multivariate COX regression analysis (*p* < 0.001, Table 2). Regarding the prognosis of PC patients with diabetes mellitus, GLMI (AUC = 0.721) demonstrated greater efficacy in predicting prognostics than TyG, TG/HDL‐c, and TyG‐BMI. It will be interested to investigate the association between GLMI and blood glucose level, IR and outcomes of PC patients with diabetes in large‐scale, prospective studies.

In summary, we found IR was an unfavorable factors for PC patients with diabetes mellitus. GLMI, a simple and practical indicator, may reflect the degree of IR and have a good predictive value for the prognosis of PC patients with diabetes mellitus.

### 4.1 Limitations of this study

This study is a single‐center retrospective study with a relatively limited sample size, which may lead to a certain selection bias and a class I error. Moreover, the study includes PC patients with various pathological types, resulting in a certain degree of heterogeneity. Additionally, diabetes mellitus plays a multifaceted role in PC, and this study only examines IR as a manifestation of diabetes mellitus, which is not comprehensive enough.

## AUTHOR CONTRIBUTIONS

**Hailiang Wang:** Conceptualization (equal); data curation (equal); writing – original draft (equal). **Shiye Ruan:** Data curation (equal); software (equal). **Zelong Wu:** Data curation (equal); resources (equal). **Qian Yan:** Formal analysis (equal); software (equal). **Yubin Chen:** Formal analysis (equal); software (equal). **Jinwei Cui:** Data curation (equal); resources (equal). **Zhongyan Zhang:** Data curation (equal); resources (equal). **Shanzhou Huang:** Project administration (equal); writing – review and editing (equal). **Baohua Hou:** Project administration (equal); writing – review and editing (equal). **Chuanzhao Zhang:** Conceptualization (equal); writing – review and editing (equal).

## FUNDING INFORMATION

This study was supported by National Natural Science Foundation of China (82072635 and 82072637), Special Events Supported by Heyuan People's Hospital (YNKT202202), the Science and Technology Program of Heyuan (23051017147335), Funding of Guangdong Provincial People's Hospital (KY012021164), Funding of Weihai Central Hospital (2023KY‐02).

## CONFLICT OF INTEREST STATEMENT

The authors have no conflict of interest.

## ETHICS STATEMENT

The study protocols was performed strictly according to the Declaration of Helsinki and was approved by the ethics committee of Guangdong Provincial People's Hospital. Written informed consent was obtained from each patient for medical record review and data analysis in this study.

## Data Availability

The data that support the findings of this study are available from the corresponding author upon reasonable request.
